# Evaluation of Bone Metabolism Biomarkers in Paget's Disease of Bone

**DOI:** 10.7759/cureus.4791

**Published:** 2019-05-31

**Authors:** Glaucio R Werner de Castro, Ziliani Da Silva Buss, Julia Salvan Rosa, Bruno M Facchin, Tania S Fröde

**Affiliations:** 1 Rheumatology, University of Southern Santa Catarina, Florianopolis, BRA; 2 Clinical Analysis, Federal University of Santa Catarina, Florianopolis, BRA

**Keywords:** paget's disease of bone, osteopontin, sclerostin, rankl, osteoprotegerin, dkk-1, sfrp-1

## Abstract

Objective: To evaluate serum levels of bone metabolism biomarkers in patients with Paget's disease of bone (PDB).

Methods: Serum levels of osteopontin, sclerostin, receptor activator of nuclear factor kappa-Β ligand (RANKL), osteoprotegerin, Dickkopf-related protein 1 (DKK-1), and soluble frizzled-related protein 1 (sFRP-1) were measured in 57 patients with Paget’s disease of bone and 24 controls with primary osteoarthritis. Subgroup analysis was employed to identify any differences in bone metabolism biomarker levels according to disease activity or current treatment.

Results: Patients with PDB presented higher levels of osteopontin and RANKL. When compared with patients with inactive disease, patients with active disease presented higher levels of bone-specific alkaline phosphatase (BAP) and osteopontin. There was a significant correlation between serum levels of BAP and osteopontin. There was no significant correlation between levels of BAP and other bone metabolism biomarkers. Current disease extension on bone scintigraphy had a significant correlation with serum levels of osteopontin and BAP. There was no significant correlation between current disease extension and other bone metabolism biomarkers. Serum levels of osteopontin and RANKL were correlated to serum levels of BAP and disease extension.

Conclusion: Patients with PDB presented higher levels of osteopontin and RANKL. Osteopontin could be a useful biomarker for activity and extension of PDB.

## Introduction

Paget’s disease of bone (PDB) is a common osteometabolic disease characterized by increased and disorganized bone turnover. It is usually asymptomatic, but some patients may present with bone pain, fractures, deformities, secondary osteoarthritis, neurologic and cardiac complications and, in rare cases, neoplasm. It is believed that PDB is caused by alterations in the behavior of osteoclasts since pagetic bone is rich in overactive osteoclasts, and medications that act in these cells, as bisphosphonates, are very effective in PDB [1, 2].

In physiologic conditions, activation of osteoclasts and osteoblasts is tightly coordinated to assure that all reabsorbed bone by osteoclasts will be replaced by new bone made by osteoblasts. Osteoblasts regulate osteoclasts secreting mediators as RANKL and osteoprotegerin; osteoclasts on the other hand, stimulate osteoblasts' differentiation and activation by secretion of cytokines, the liberation of growth factors previously embedded in the bone matrix and by cell to cell contact. Osteocytes are key sensors that regulate both osteoclasts and osteoblasts, secreting, among other mediators, prostaglandins, RANKL, and sclerostin [3, 4].

The interaction between these cells in pathological conditions such as PDB remains to be elucidated. Evaluation of bone metabolism biomarkers can not only clarify some aspects of the pathogenesis of PDB but also provide new instruments to evaluate disease activity and response to treatment.

This study was undertaken to compare serum levels of the bone metabolism biomarkers RANKL, osteoprotegerin, sclerostin, Dickkopf-related protein-1 (DKK-1), soluble frizzled-related protein 1 (sFRP-1) and osteopontin in PDB patients and controls with bone metabolic diseases and to identify factors associated with any detected differences.

## Materials and methods

The methodology of this work was already partly published elsewhere [5].

Consecutive patients with PDB followed by rheumatologists in Florianopolis, Brazil were included after signing an informed consent form. Patients with primary osteoarthritis followed at the Rheumatology Outpatient Clinic of Hospital Governador Celso Ramos from the same age group (within up to five years difference from a PDB patient) were recruited as controls. Patients with primary osteoarthritis were chosen as controls because of the high prevalence of this disease in the age group affected by PDB; most patients with PDB have concomitant primary osteoarthritis. For both patients and controls the exclusion criteria were systemic inflammatory diseases, active infections, neoplasms, chronic kidney disease, malabsorption syndromes, parathyroid disorders, obesity, uncompensated hypothyroidism, alterations of serum levels of calcium, phosphorus and magnesium, erosive osteoarthritis and current use of any of the drugs known to affect bone metabolism, including, corticosteroids, with the exception of calcium and vitamin D. The patients were allowed to take bisphosphonates or calcitonin for the treatment of PDB.

PDB and osteoarthritis were diagnosed by characteristic findings on X-rays. Disease activity was evaluated by 99mTc MDP bone scintigraphy; a patient was considered to have active disease when the bone scan shows high uptake suggestive of PDB, and other possible diagnoses were excluded by X-rays or other imaging techniques. All high uptake areas were evaluated by X-rays or computed tomography. The disease extent was determined by previous and recent X-rays and bone scintigraphy; the method described by Meunier et al. was used to calculate disease extent on bone scintigraphy [6]. A patient was considered to be at current treatment if he had used oral bisphosphonates (alendronate, risedronate or ibandronate) in the past six months or zoledronic acid in the previous 12 months. No patients had taken calcitonin, intravenous ibandronate or pamidronate over the last 12 months.

Fasting blood samples were collected from patients and controls with primary osteoarthritis for determination, by enzyme-linked immunosorbent assay, of serum levels of osteopontin (Abcam, Cambridge, MA, ref. AB100618), sclerostin (Mybiosource, San Diego, CA, ref. MBS702938), RANKL (BioVendor, Czech Republic, ref. RD193004200R), osteoprotegerin (Raybiotech Inc. Norcross, GA, ref. ELH-OPG-001), DKK-1 (Raybiotech Inc. Norcross, GA, ref. ELH-DKK1-001), and sFRP-1 (Mybiosource, San Diego, CA, MBS705581). Bone-specific alkaline phosphatase (BAP) (Mybiosource, San Diego, CA, ref. MBS724100) was also measured in the serum of PDB patients. For these analyses, serum levels of cytokines in PDB patients were compared to the results of controls with primary osteoarthritis. In order to identify factors possibly associated with cytokine alterations, PDB patients were further subdivided according to disease activity and treatment status. We also searched for correlations between serum bone metabolism biomarkers and disease extent and BAP levels. Patients who received treatment with zoledronic acid during the study period were submitted to a new blood collection three months after the infusion.

Results were presented as mean (SD) or percentage. Comparisons between groups were made with analysis of variance (ANOVA). Correlations between linear variables were analyzed by Pearson's correlation. In all cases, a curve-fitting procedure with the models linear, exponential, logistic, logarithmic, cubic and quadratic was employed to evaluate if the correlation between two variables could fit better in a nonlinear model. Repeated measures were analyzed with paired samples T-test. Statistical analysis was performed with SPSS Statistics for Windows, Version 20.0 (IBM Corp. Armonk, NY), with a level of significance of 0.05.

The study protocol was approved by the local ethical committee (protocol number 353461). This study was conducted according to the principles of the Declaration of Helsinki (“WMA Declaration of Helsinki - Ethical Principles for Medical Research Involving Human Subjects,” n.d.) [7].

## Results

Fifty-seven patients (mean age 66.8±9.3 years, 64.9% females) and 24 controls with primary osteoarthritis (mean age 62.4±6.9 years, 83.3% females) were included in the study. Patients had a mean time from diagnosis of 8.51±6.3 years; 38.6% had a monostotic disease, and 31.6% were considered to be in current treatment for PDB. Familial history of PDB was detected in 31.5%, mutations of SQSTM-1 gene were present in 19.6% of the patients.

**Figure 1:**
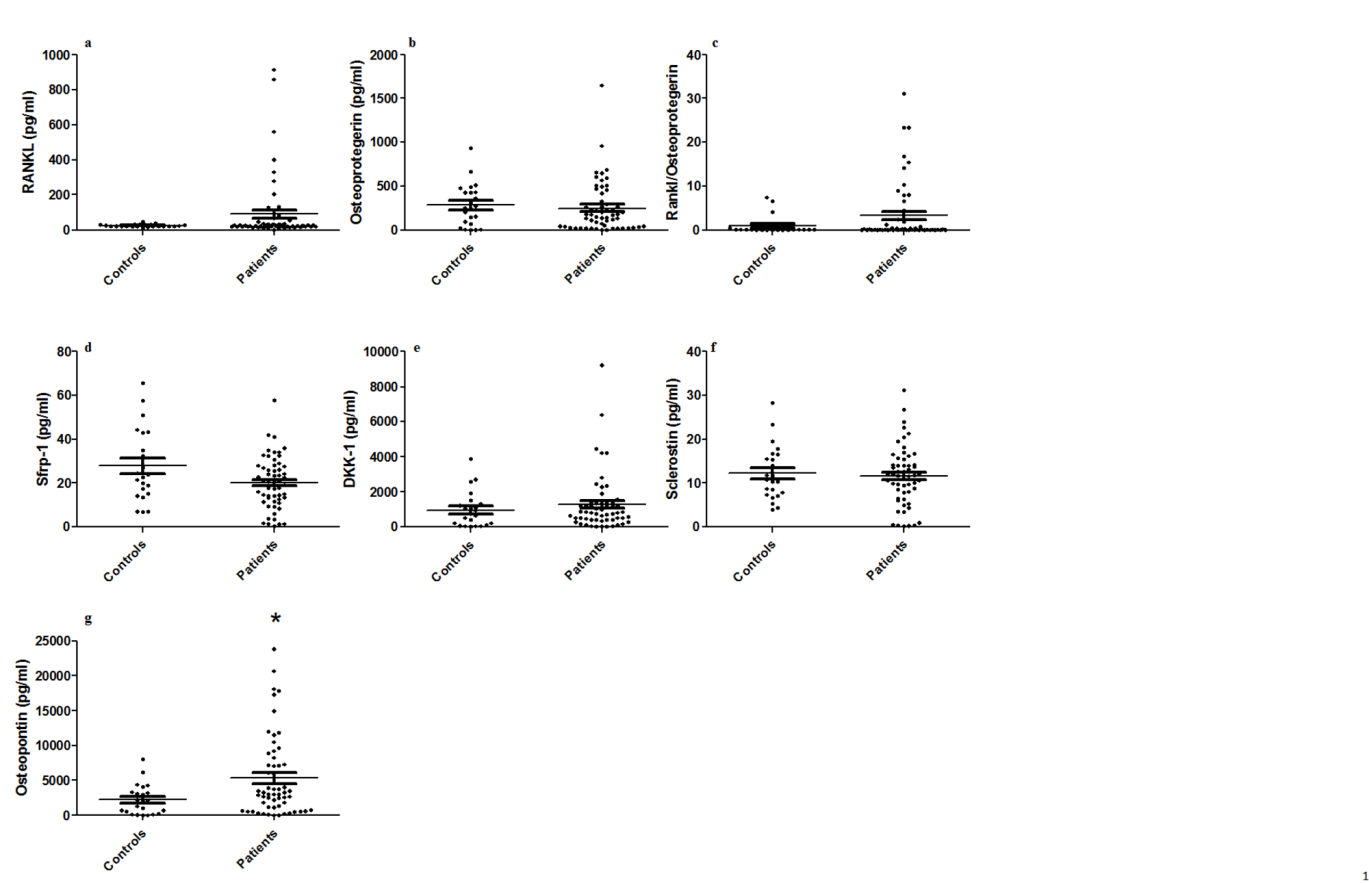
Dosage of serum bone metabolism biomarkers (a) RANKL, (b) osteoprotegerin, (c) RANKL / osteoprotegerin ratio, (d) DKK-1, (e) sFRP-1, (f) sclerostin, and (g) osteopontin in patients with PDB and controls. Each dot represents the results of an individual patient. Lines represent mean, intervals represent SD. * = p<0.05, comparisons were done with ANOVA. DKK-1 - Dickkopf-related protein-1, sFRP-1 - soluble frizzled-related protein 1, PDB - Paget’s disease of bone, ANOVA - analysis of variance

There were no statistically significant differences between patients and controls with primary osteoarthritis in serum levels of sclerostin (11.51±6.74 pg/mL vs 12.24±6.20 pg/mL, p = 0.992), DKK-1 (1280.14±1644.05 pg/mL vs 955.61±1007.63 pg/mL, p = 0.847), sFRP-1 (24.33±33.79 pg/mL vs 27.78±16.67 pg/mL, p = 0.951), and osteoprotegerin (249.63±292.25 pg/mL vs 281.71±241.34 pg/mL, p = 0.969). Although the difference had not reached statistical significance, levels of serum RANKL were higher in patients in comparison to controls (110.27±214.40 pg/mL vs 24.69±8.00 pg/mL, p = 0.061), as were the relations between serum levels of RANKL and osteoprotegerin (4.92 ±14.10 vs 0.97±2.14, p = 0.195). Patients with PDB presented significantly higher levels of osteopontin (6724.19±6366.32 pg/mL vs 2201.33±2156.49 pg/mL, p = 0.002) (Figure 1).

Because levels of biomarkers of bone metabolism could differ between patients with active and with inactive disease, we made a comparison between patients with active disease (n=39) and controls, but this approach revealed a similar pattern to the comparison between controls and the whole group of patients (Table 1).

**Table 1:** Comparison of serum levels of bone metabolism biomarkers in patients with PDB and in controls with primary osteoarthritis. RANKL - receptor activator of nuclear factor kappa-Β ligand, DKK-1 - Dickkopf-related protein-1, sFRP-1 - soluble frizzled-related protein 1, PDB - Paget’s disease of bone, OPG - osteoprotegerin

	Controls (n = 24)	PDB with active disease (n = 39)	p value
RANKL (pg/ml)	24.69 (8.00)	110.27 (214.40)	p = 0.061
Osteoprotegerin (pg/ml)	281.71(241.34)	278.60 (328.49)	p = 0.969
DKK-1 (pg/ml)	965.61(1007.63)	916.98 (921.26)	p = 0.847
sFRP-1 (pg/ml)	27.28 (16.67)	27.23 (40.07)	p = 0.951
Sclerostine (pg/ml)	12.24 (6.20)	12.25 (6.80)	p = 0.992
Osteopontin (pg/ml)	2201.33 (2156.49)	6724.19 (6366.32)	p = 0.002
RANKL/Opg	0.973 (2.14)	3.42 (7.06)	p = 0.119

To investigate if the levels of bone metabolism biomarkers were influenced by PDB activity, we compared subgroups of patients divided according to disease activity and to current treatment. We also searched for correlations between cytokine levels and disease extent and BAP levels. When patients were compared according to the presence of areas of high uptake on bone scintigraphy, those with active (n = 39) presented higher levels of BAP (p = 0.032) and osteopontin (p = 0.006), but lower levels of DKK-1 (p = 0.011). There were no statistically significant differences in the levels of other biomarkers (p>0.05) (Table 2). There were no statistically significant differences in bone metabolism biomarker levels between patients in current treatment (n = 18) and patients without current treatment (p>0.05) (Table 2).

**Table 2:** Comparison of serum levels of biomarkers of bone metabolism in patients with PDB according to disease activity and current treatment (defined as use of oral bisphosphonates in the last six months or zoledronic acid in the last 12 months). RANKL - receptor activator of nuclear factor kappa-Β ligand, DKK-1 - Dickkopf-related protein-1, sFRP-1 - soluble frizzled-related protein 1, PDB - Paget’s disease of bone, OPG - osteoprotegerin, BAP - bone-specific alkaline phosphatase

		Disease activity				Current treatment	
	Active (n = 39)		Inactive (n = 18)	p value	Yes (n = 18 )	No (n = 39)	p value
RANKL (pg/ml)	110.27 (214.40)		158.83 (481.29)	p = 0.598	69.66 (196.66)	151.43 (361.79)	p = 0.374
Osteoprotegerin (pg/ml)	278.60 (328.49)		168.88 (184.55)	p = 0.275	250.42 (202.24)	249.27 (327.98)	p = 0.989
DKK-1 (pg/ml)	916.98 (921.26)		2113,.7 (2493.30)	p = 0.011	964.74 (739.30)	1429.54 (1922.34)	p = 0.328
sFRP-1 (pg/ml)	27.23 (40.07)		18.06 (10.56)	p = 0.346	23.95 (11.94)	24.51 (40.23)	p = 0.955
Sclerostine (pg/ml)	12.25 (6.80)		9.80 (6.48)	p = 0.214	13.29 (6.88)	10.47 (6.51)	p = 0.096
Osteopontin (pg/ml)	6724,19 (6366,32)		2312,92 (2183,22)	p = 0,006	5922,56 (1395,96)	5347,27(5768,54)	p = 0,976
RANKL/Opg	3.42 (7.06)		8.12 (22.78)	p = 0,.48	2.23 (7.52)	6,.1( 16,10)	p = 0,348
BAP (U/l)	59.56 (76.20)		19.77 (7.76)	p = 0.032	28.59 (18.38)	55.38 (77.23)	p = 0.154

**Figure 2:**
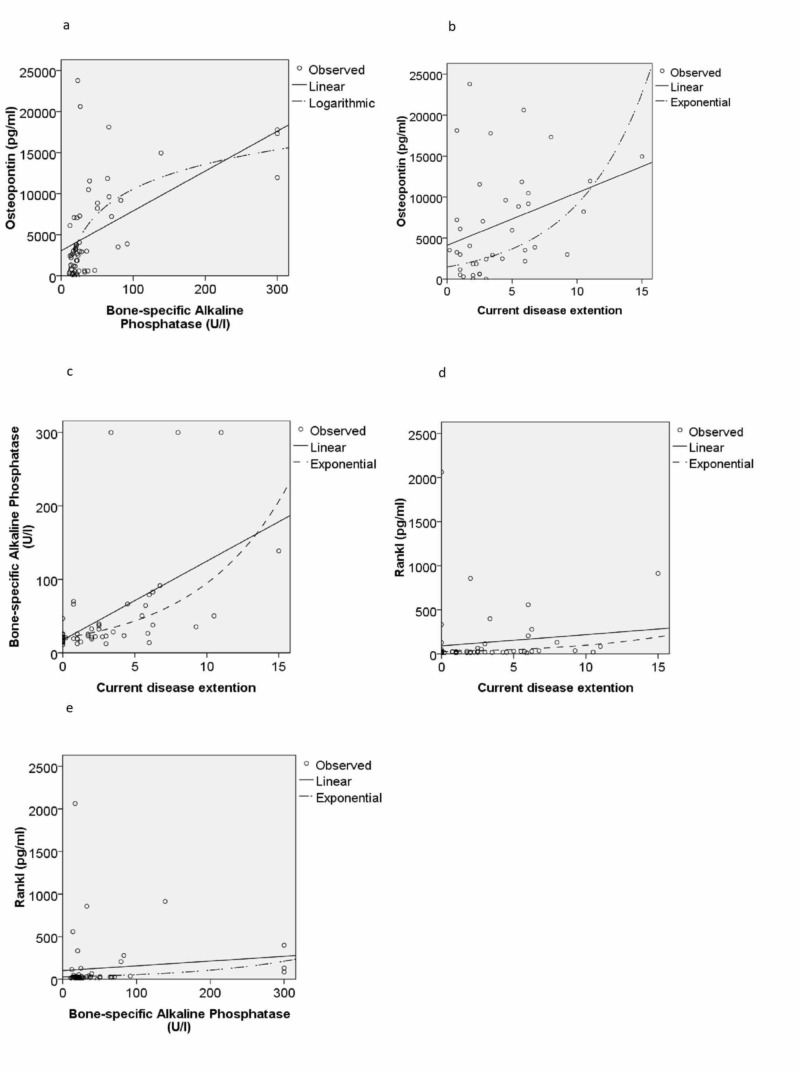
Correlation between serum levels of (a) osteopontin and bone-specific alkaline phosphatase, (b) osteopontin and current disease extent, (c) bone-specific alkaline phosphatase and current disease extent, (d) RANKL and current disease extent and (e) RANKL and bone-specific alkaline phosphatase in 57 patients with PDB. Each point represents the results of an individual patient. Lines represent results of regressions: linear, logarithmic or exponential. RANKL - receptor activator of nuclear factor kappa-Β ligand, PDB - Paget's disease of bone

There was a significant correlation between serum levels of BAP and osteopontin (r 0.549 p <0.001). There was no significant correlation between levels of BAP and other bone metabolism biomarkers (DKK-1: r = 0.065 p = 0.636, sFRP1: r = -0.033 p = 0.809, sclerostin: r = -0.124 p = 0.368), RANKL r = 0.113 p = 0.405 and osteoprotegerin (r = -0.140 p = 0.304) (Figure 2).

**Figure 3:**
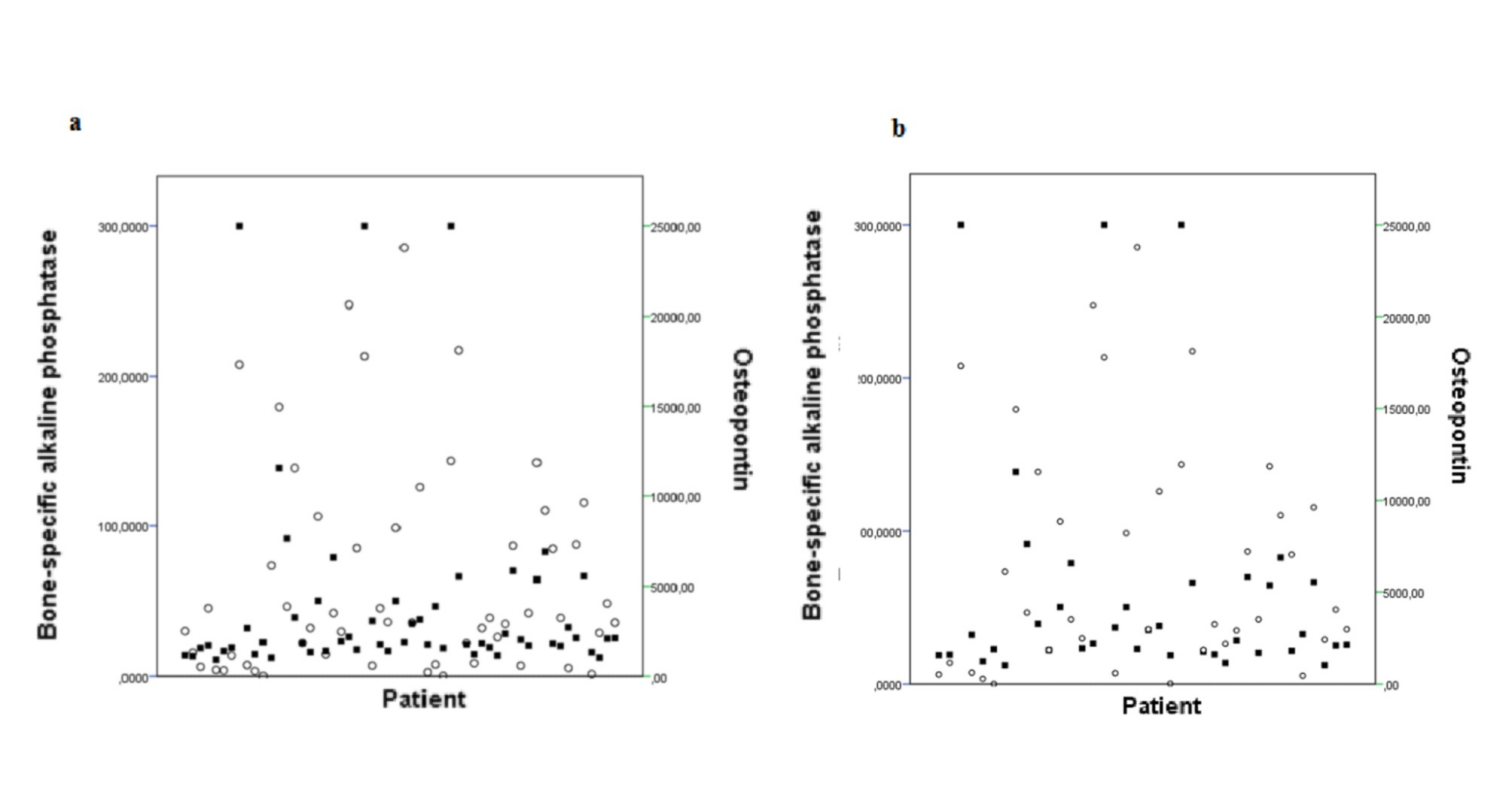
Serum concentratrions of BAP (squares) and osteopontin (circles) in all patients (a) and only in patients with active disease at bone scintigraphy (b). Each column represents a patient. BAP - bone-specific alkaline phosphatase

Current disease extent on bone scintigraphy had a significant correlation with serum levels of osteopontin (r = 0.449 p = 0.001) and BAP (r = 0.547 p <0.001) (Figure 3). There was no significant correlation between current disease extent and other bone metabolism biomarkers (DKK-1: r = -0.206 p = 0.131, sFRP-1: r= 0.045 p = 0.743, sclerostin r = -0.29 p = 0.831, RANKL: r = 0.132 p = 0.330 and osteoprotegerin r = -0.023 p = 0.868). In spite of the correlation between BAP and osteopontin, some patients presented discordant results (Figure 3).

**Figure 4:**
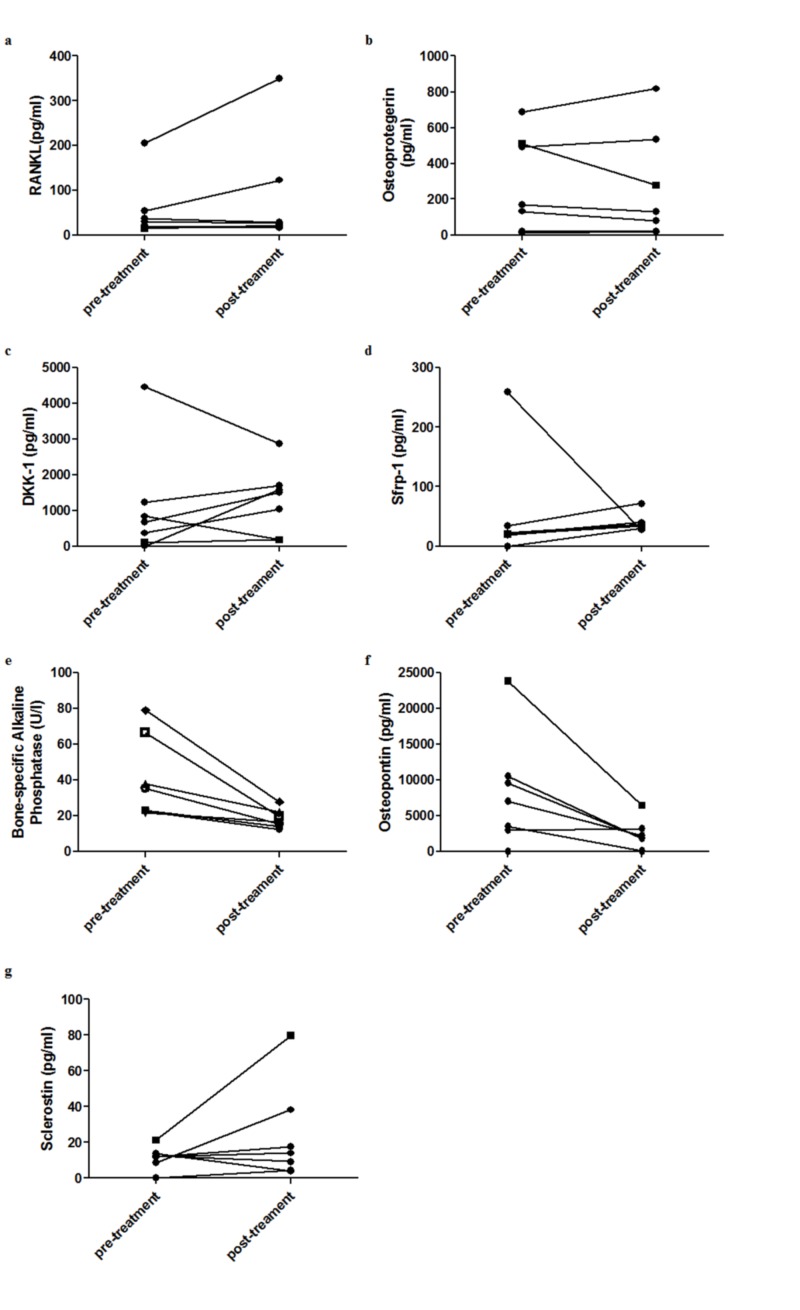
Dosage of serum bone metabolism biomarkers (a) RANKL, (b) osteoprotegerin, (c) DKK-1, (d) sFRP-1, (e) osteopontin, (f) bone-specific alkaline phosphatase and (g) sclerostin in seven patients with PDB pre-treatment with zoledronic acid and after three months. Each line represents the results of an individual patient. RANKL - receptor activator of nuclear factor kappa-Β ligand, DKK-1 - Dickkopf-related protein-1, sFRP-1 - soluble frizzled-related protein 1, PDB - Paget’s disease of bone

To evaluate if the levels of biomarkers of bone metabolism could be related to BAP levels or disease extent in a non-linear manner, we ran a curve-fitting procedure. It confirmed the correlation between serum levels of osteopontin and BAP and between levels of both biomarkers and the disease extent at the bone scintigraphy. However, the model that best fitted the correlation between osteopontin and BAP was the logarithmic model (r = 0.599 p <0.001). On the other hand, the correlation between BAP levels and disease extent was better expressed by the exponential model (r = 0.659 p <0.001). Additionally, these models have revealed that serum levels of RANKL presented a significant correlation in exponential model to current disease extent (r = 0.327 p = 0.014) and to BAP levels (r = 0.324 p = 0,015) (Figure 4). There was no significant correlation between BAP or current disease extent and the remaining bone metabolism biomarkers in any of the models. During the study, seven patients with active PDB were treated with zoledronic acid 5 mg and had their cytokines and BAP levels measured before and three months after treatment. After the treatment, there were reductions in values of osteopontin (8208.52±7829.00 pg/mL vs 2263.19±2163.47 pg/mL p = 0,041) and BAP (34.51±17.14 U/L vs 16.67±.3.56 U/L p = 0.035). The other bone metabolism biomarkers presented less intense variations, the meaning of which is difficult to be interpreted because of the small number of patients (DKK-1: 1101.52±1541.96 pg/mL vs 1293.21±940.27 pg/mL, sFRP1: 53.75±91.18 pg/mL vs 39.80±14.804 pg/mL, sclerostin: 11.52±6.32 pg/mL vs 23.48±27.45 pg/mL, RANKL: 54.57±68.14 pg/mL vs 83.55±123.77 pg/mL, osteoprotegerin 289.29±269.85 pg/mL vs 269.15±303.43 pg/mL) (Figure 4).

## Discussion

The advent of powerful intravenous bisphosphonates such as zoledronic acid has been a significant advance in the treatment of PDB, providing effective disease control for most patients. Besides, as these drugs act primarily in osteoclasts, it has confirmed the role of these cells in the pathogenesis of PDB.

However, many aspects of PDB's pathogenesis remain to be elucidated, including the complex relationship between the osteoclasts' hyperactivity and the consequent activation of osteoblasts, which results in the characteristic disorganized bone, and the role of osteocytes in this disease.

RANKL has critical functions in the differentiation and activation of osteoclasts. In physiological conditions, its main sources are osteoblasts and osteocytes, which also secrete its inhibitor, osteoprotegerin [8]. The net effect on osteoclasts and its progenitors depends on the balance between the secretion of RANKL and osteoprotegerin. Typically, pagetic bone presents a high number of osteoclasts, which are enlarged, hyperactive and hyper-responsive to RANKL [9]. Moreover, hyperexpression of RAKNL in bone marrow cells from pagetic tissue has been demonstrated [10]. Considering these facts, it would be tempting to imagine that serum RANKL levels should be high in PDB patients. However, many factors could contribute to making this untrue: hyper-responsive osteoclasts need, in fact, lower levels of RANKL to be activated; many effects of RANKL on osteoclasts occur in cell to cell contact or in a paracrine way; and the plasmatic pool of RANKL and osteoprotegerin may come from additional sources, beyond bone tissue [11].

In the present study, osteoprotegerin levels in patients did not differ significantly from controls; although the difference did not reach statistical significance, patients showed higher levels of RANKL than controls. Moreover, RANKL levels were correlated with disease extent and BAP levels. There is a considerable divergence between previous studies regarding RANKL and osteoprotegerin levels in PDB. Alvarez at al. [12] did not find significant differences in RANKL levels between patients and controls but reported that PDB patients present higher levels of osteoprotegerin. In line with the current study, they described that levels of RANKL and osteoprotegerin did not change significantly with the use of bisphosphonates, a finding also reported by Polyzos et al. [13]. Martini et al. [14] found higher levels of RANKL and osteoprotegerin in PDB and reported increasing of osteoprotegerin levels after treatment, a finding also described by Mossetti et al. [15]. Additional studies will be necessary to clarify if PDB causes alterations in serum levels of RANKL and osteoprotegerin as well as their clinical significance since bone levels are probably more important.

The Wnt/b-catenin signaling pathway is one of the most critical factors for the differentiation of osteoblasts. Wnt glycoproteins may be synthesized by many kinds of cells, including osteoclasts, whose differentiation could also be influenced by these mediators [16-18]. Wnt glycoproteins are poorly soluble and, hence, difficult to be measured in serum samples. On the other hand, its inhibitors are measurable, including sclerostin, mainly produced by osteocytes, DKK-1 and sFRP-1, both produced by many kinds of cells, including osteocytes [19-21]. In PDB, the excessive bone resorption results in an equally excessive osteoblast activation. Presumably, osteoblast activation involves signaling through the Wnt/beta-catenin pathway. Theoretically, levels of the Wnt/b-catenin pathway could be diminished, thus contributing to the activation of osteoblasts, or even elevated, as an answer to the excess of osteoblast activation.

In the present study, levels of sclerostin, DKK-1, and sFRP-1 in the serum of PDB patients did not differ from controls. Besides, serum levels of sFRP-1 and sclerostin did not differ in patients with active or inactive disease and, along with DKK-1, were not significantly altered with treatment. DKK-1 levels were higher in patients with active disease, but this observation should be interpreted with caution because it can be due to chance. This hypothesis is reinforced by the lack of significant reduction alteration in DKK-1 levels with treatment. To the best of our knowledge, sFRP-1 levels in PDB have not been reported in any previous study. With regard to DKK-1, Polyzos et al. [13] did not find differences between patients and controls, as in the present study. However, Marshall et al. [22] reported higher levels of DKK-1 in PDB patients, although there were no differences between treated and untreated patients. Sclerostin levels in PDB have been reported by only one study before. Yavropoulou et al. [23] have found higher levels of sclerostin in PDB, which differs from the findings of the present study.

It should be emphasized that the absence of an alteration in serum levels of the inhibitors of the Wnt/beta-catenin pathway does not rule out a role of this signaling system in PDB pathogenesis. In fact, if any Wnt glycoprotein is elevated in PDB tissue, the absence of elevation of its inhibitors could mean a relative deficiency of inhibitors. Moreover, many tissues beyond bone produce these mediators, so serum levels do not reflect only bone production. Additional studies, ideally with the pagetic bone itself, will be necessary to elucidate the role of the Wnt/beta-catenin pathway in PDB.

Osteopontin is expressed in many tissues and has varied functions, including cell adhesion, chemotaxis, control of cell growth, and regulation of the immune system [24-26]. In bone, it is produced mainly by osteoblasts, and it is one of the main components of the extracellular matrix. It has important functions in bone, particularly in the regulation of nucleation and growing of hydroxyapatite crystals and in cell adhesion. It can bind to hydroxyapatite crystals and to integrins expressed in cellular membranes that, like CD44, work as receptors for the signaling functions of this protein. Beyond these roles, it allows the anchorage of bone cells to the bone matrix and is essential to osteoclast migration [27, 28].

In the present study, it has been demonstrated that patients with PDB exhibit higher serum levels of osteopontin in comparison to controls. Moreover, those with active disease have higher levels than the patients with inactive disease, serum concentrations of osteopontin have a positive correlation with the disease extent and BAP and are reduced after treatment with zoledronic acid. These data indicate that osteopontin production seems to be increased in bones affected by PDB and suggest that it could have a role as a biomarker for the activity of PDB. To the best of our knowledge, this is the first study to describe osteopontin levels in PDB.

Some patients with active PDB present normal levels of BAP. This could be a problem for both disease diagnosis and treatment monitoring. Interestingly, some patients with areas of high uptake on bone scintigraphy present discordant levels of BAP and osteopontin (Figure 3b).

The present study has some limitations. In clinical practice, the usual method to evaluate PDB activity is the serum alkaline phosphatase level. We were not able to assess disease activity with this approach because we chose to measure bone-specific alkaline phosphatase by an enzyme-linked immunosorbent assay (ELISA). This assay is not used in clinical practice. Hence, we do not have a cutoff for normal levels. However, some patients with PDB may present with bone pain and altered bone scintigraphy while with serum alkaline phosphatase level in the normal range. These patients usually get relief from their symptoms after treatment with zoledronic acid. This means that not every patient with active PDB can be identified only by high alkaline phosphatase levels. Another limitation that should be taken into account while interpreting the results is the low number of subjects in subgroup analyses. However, the results are consistent across all analyses, which means the probability that the results are due to chance is low.

Another possible point of concern might be the option to use subjects with osteoarthritis as controls. This is justified because osteoarthritis is very prevalent in the age group affected by PDB [29, 30]. Therefore, it is challenging to find people aged over 60 years without osteoarthritis. Besides, many of those who do not have osteoarthritis have other prevalent diseases in this age group, such as obesity, diabetes mellitus, neoplasm, heart diseases. Most of the elderly people with osteoarthritis are asymptomatic and, in fact, could be considered “healthy” for most studies. It should be emphasized that the majority of our controls were asymptomatic, although they have radiographic evidence of osteoarthritis, and that many of our PDB patients also have primary osteoarthritis.

## Conclusions

In conclusion, serum osteopontin levels could be a useful biomarker in PDB: it is increased in patients with active disease, it is correlated with the disease extent and is altered by the treatment. Additional studies comparing its performance with alkaline phosphatase, currently the preferred method for evaluation of PDB activity, are warranted.

## References

[1] Ralston SH (2008). Pathogenesis of Paget’s disease of bone. Bone.

[2] Werner de Castro GR, Heiden GI, Zimmermann AF (2012). Paget’s disease of bone: analysis of 134 cases from an island in Southern Brazil: another cluster of Paget’s disease of bone in South America. Rheumatol Int.

[3] Matsuo K, Otaki N (2012). Bone cell interactions through Eph/ephrin. Cell Adh Migr.

[4] O’Brien CA, Nakashima T, Takayanagi H (2013). Osteocyte control of osteoclastogenesis. Bone.

[5] Werner de Castro GR, Buss Z, Rosa JS, Frode TS (2014). Inflammatory cytokines in Paget's disease of bone. Int Immunopharmacol.

[6] Meunier PJ, Salson C, Mathieu L, Chapuy MC, Delmas P, Alexandre C, Charhon S (1987). Skeletal distribution and biochemical parameters of Paget’s disease. Clin Orthop Relat Res.

[7] (2019). WMA Declaration of Helsinki - Ethical Principles for Medical Research Involving Human Subjects. World Medical Association.

[8] Komori T (2013). Functions of the osteocyte network in the regulation of bone mass. Cell Tissue Res.

[9] Neale SD, Smith R, Wass JA, Athanasou NA (2000). Osteoclast differentiation from circulating mononuclear precursors in Paget’s disease is hypersensitive to 1,25-dihydroxyvitamin D(3) and RANKL. Bone.

[10] Menaa C, Reddy SV, Kurihara N (2000). Enhanced RANK ligand expression and responsivity of bone marrow cells in Paget’s disease of bone. J Clin Invest.

[11] Dovio A, Data V, Angeli A (2005). Circulating osteoprotegerin and soluble RANKL: do they have a future in clinical practice?. J Endocrinol Invest.

[12] Alvarez L, Peris P, Guañabens N (2003). Serum osteoprotegerin and its ligand in Paget’s disease of bone: relationship to disease activity and effect of treatment with bisphosphonates. Arthritis Rheum.

[13] Polyzos SA, Anastasilakis AD, Efstathiadou Z (2009). The effect of zoledronic acid on serum dickkopf-1, osteoprotegerin, and RANKL in patients with Paget’s disease of bone. Horm Metab Res.

[14] Martini G, Gennari L, Merlotti D (2007). Serum OPG and RANKL levels before and after intravenous bisphosphonate treatment in Paget’s disease of bone. Bone.

[15] Mossetti G, Rendina D, De Filippo G (2005). Interleukin-6 and osteoprotegerin systems in Paget’s disease of bone: relationship to risedronate treatment. Bone.

[16] Pederson L, Ruan M, Westendorf JJ, Khosla S, Oursler MJ (2008). Regulation of bone formation by osteoclasts involves Wnt/BMP signaling and the chemokine sphingosine-1-phosphate. Proc Natl Acad Sci USA.

[17] Qiang Y-W, Chen Y, Brown N, Hu B, Epstein J, Barlogie B, Shaughnessy JD Jr. (2010). Characterization of Wnt/beta-catenin signalling in osteoclasts in multiple myeloma. Br J Haematol.

[18] Glass DA 2nd, Bialek P, Ahn JD (2005). Canonical Wnt signaling in differentiated osteoblasts controls osteoclast differentiation. Dev Cell.

[19] Burgers TA, Williams BO (2013). Regulation of Wnt/β-catenin signaling within and from osteocytes. Bone.

[20] Maeda K, Takahashi N, Kobayashi Y (2013). Roles of Wnt signals in bone resorption during physiological and pathological states. J Mol Med.

[21] Bonewald LF, Johnson ML (2008). Osteocytes mechanosensing and Wnt signaling. Bone.

[22] Marshall MJ, Evans SF, Sharp CA, Powell DE, McCarthy HS, Davie MWJ (2009). Increased circulating Dickkopf-1 in Paget’s disease of bone. Clin Biochem.

[23] Yavropoulou MP, van Lierop AH, Hamdy NAT, Rizzoli R, Papapoulos SE (2012). Serum sclerostin levels in Paget’s disease and prostate cancer with bone metastases with a wide range of bone turnover. Bone.

[24] Shin T (2012). Osteopontin as a two-sided mediator in acute neuroinflammation in rat models. Acta Histochemica.

[25] Haylock DN, Nilsson SK (2006). Osteopontin: a bridge between bone and blood. Br J Haematol.

[26] Das R, Philip S, Mahabeleshwar GH, Bulbule A, Kundu GC (2005). Osteopontin: its role in regulation of cell motility and nuclear factor κB-mediated urokinase type plasminogen activator expression. IUBMB Life.

[27] Kazanecki CC, Uzwiak DJ, Denhardt DT (2007). Control of osteopontin signaling and function by post-translational phosphorylation and protein folding. J Cell Biochem.

[28] McKee MD, Pedraza CE, Kaartinen MT (2011). Osteopontin and wound healing in bone. Cells Tissues Organs.

[29] Arden N, Nevitt MC (2006). Osteoarthritis epidemiology. Best Pract Res Clin Rheumatol.

[30] van Saase JL, van Romunde LK, Cats A, Vandenbroucke JP, Valkenburg HA (1989). Epidemiology of osteoarthritis Zoetermeer survey. Comparison of radiological osteoarthritis in a Dutch population with that in 10 other populations. Ann Rheum Dis.

